# Comparative Analysis of Lower Back Pain and Phantom Pain in Unilateral Lower Limb Amputees: A Study on Amputee Football Players vs. Non-Athletes

**DOI:** 10.3390/medicina60122047

**Published:** 2024-12-12

**Authors:** Aleksandra Jóźwik, Agnieszka Wnuk-Scardaccione, Jan Bilski

**Affiliations:** 1 Graduate of Physiotherapy, Faculty of Health Sciences, Jagiellonian University Medical College, 8 Skawińska Street, 31-066 Krakow, Poland; a.jozwik@student.uj.edu.pl; 2 Department of Biomechanics and Kinesiology, Institute of Physiotherapy, Faculty of Health Sciences, Jagiellonian University Medical College, 8 Skawińska Street, 31-066 Krakow, Poland; agnieszka90.wnuk@uj.edu.pl

**Keywords:** amputation, low back pain, amputee football

## Abstract

*Background and Objectives*: Amputation poses a significant clinical and therapeutic challenge, with over 90.0% of amputations involving the lower limbs, of which 75.0% are associated with diabetes and peripheral artery disease. Individuals with lower limb amputations often experience secondary disabilities. This study aimed to compare the frequency and intensity of lower back pain and phantom pain in individuals with unilateral lower limb amputations who were amputee football players with those who did not participate in adaptive sports. *Materials and Methods*: This study included 30 men who had undergone unilateral lower limb amputation and were divided into two groups: 15 amputee football players and 15 men who did not participate in adapted sports. Testing included proprietary surveys, questionnaires, the Oswestry Disability Index (ODI), and the visual analog scale (VAS) for pain intensity. The Amputee Mobility Predictor (AMPnoPRO) was used to assess motor function. Statistical analysis was performed using Statistica software (Version 13, StatSoft, Inc., Tulsa, OK, USA) by applying Student’s *t*-test, the Mann–Whitney U test, correlation coefficients, and analysis of covariance (ANCOVA). Phantom pain was reported in 53.3% of individuals in the study group and in 66.7% of individuals in the control group, with average median pain intensities of 5.5 (IQR: 2) and 5.5 (IQR: 3), respectively. Lower back pain was significantly higher in the control group (86.7%) than for the football players (33.3%), with median pain intensities of 4 (IQR: 2) and 3 (IQR: 2), respectively. The median disability score was 3 (IQR: 3) in the player group and 10 (IQR: 7) in the control group. The median score in the amputee football player group was 35 (IQR: 2), while that in the control group was only 18 (IQR: 19). *Conclusions*: Amputee football training did not have a significant impact on the frequency or intensity of phantom pain but was associated with lower occurrence and intensity of pain in the lower back. The players exhibited significantly better motor function and lower levels of disability. Preoperative limb pain was a significant risk factor for phantom pain in the study group. Adaptive sports can not only support the development of motor and social skills but also contribute to reducing the severity of lower back pain, which leads to a decrease in the level of disability.

## 1. Introduction

Amputation remains a major clinical challenge despite advances in surgical techniques and postoperative care. While over 90.0% of amputations involve the lower limbs, 75.0% are linked to diabetes and peripheral artery disease. In contrast, in developing countries, trauma, infection, and cancer are the leading causes of amputation [1,2].

Trends in individual countries show a decline in the frequency of major lower limb amputations between 2010 and 2020, while the number of minor amputations has been increasing [3]. In the Netherlands, the number of amputations remains consistently low [4]. Poland, on the other hand, shows a continuous increase in the number of amputations performed [5,6]. Amputation rates vary between countries depending on socio-economic status, the age of the population, racial differences, access to healthcare and screening, and most importantly, patients’ awareness of their health condition [4,7]. The number of diabetes cases has risen from 151 million in 2000 to 536.6 million (10.5% of the global population) in 2021, with projections reaching 783.2 million (12.2%) by 2045 [8]. Studies across 21 countries show that while improvements in diabetes care have reduced major lower limb amputations, minor amputations have not significantly decreased [9,10,11].

Individuals with lower limb amputations often develop secondary disabilities, including lower back pain, phantom pain [12,13], gait disturbances, and balance disorders [14]. These secondary disabilities significantly reduce quality of life and the ability to perform daily activities [12,15]. The loss of self-care abilities often leads to dependency on others [16]. These limitations result in a loss of independence, negatively affecting physical, psychological, and social well-being, thereby lowering overall quality of life [15]. Consequently, physical activity and participation in daily tasks decrease, leading to a decline in physical fitness [14]. Studies have shown that lower back pain occurs much less frequently in non-amputees (12.0–45.0%) than in individuals with lower limb amputations (52.0–89.0%) [17], particularly among those with a sedentary lifestyle and who do not engage in sports [12]. Due to the limited number of studies assessing the multifactorial nature of this pain, it is unclear whether this difference is truly significant [12,18]. Further research is required to identify the potential factors contributing to its occurrence [12]. It is estimated that up to 85.0% of amputees experience intense episodes of neuropathic pain, known as phantom pain [19]. However, little is known about the mechanisms of this pain, its long-term effects, and its development over extended observation periods [20,21]. Despite the widespread occurrence of phantom and lower back pain, few studies have evaluated the impact of amputee football on these conditions in individuals with lower limb amputations. The dynamic movements involved in football improve posture and core strength, which helps prevent or alleviate lower back issues often caused by compensatory gait patterns. By providing both physical and psychological benefits, adaptive sports offer a holistic approach to improving the well-being of amputees.

This study aimed to compare the frequency and intensity of lower back pain and phantom pain in individuals with unilateral lower limb amputations who are amputee football players with those who do not participate in adaptive sports. We hypothesized that participation in amputee football would be associated with lower frequency and intensity of lower back pain, as well as better overall motor performance, compared to those who do not engage in sports. Analysis of the benefits of amputee football and its integration into rehabilitation programs could significantly improve and accelerate the rehabilitation process and contribute to enhancing the quality of life of individuals after amputation.

## 2. Materials and Methods

### 2.1. Study Design

In this retrospective study, a comparative analysis was conducted to examine lower back pain and phantom pain in individuals with amputations compared with amputee football players. Additionally, motor performance was compared between the two groups. The sample size was calculated a priori using G*Power software version 3.1.9.7. The following parameters were applied for the calculation: effect size (d) = 0.8, α error probability = 0.05, and power (1 − β error probability) = 0.8. After inputting all the data into the calculator, the minimum sample size per group was determined to be 14 participants.

### 2.2. Study Participants

The study included 30 men with unilateral lower limb amputations. The study group consisted of 15 amputee football players, whereas the control group consisted of 15 men with unilateral amputations who did not participate in adaptive sports. Data from the complete medical records of individuals aged 18–70 years were considered, provided they had not visited the rehabilitation center due to lower limb injury or illnesses that would prevent initial functional testing. Individuals were excluded if they had bilateral lower limb amputations; upper limb amputations; or acute cardiovascular, respiratory, or oncological conditions. Participants who had undergone surgery on the non-amputated limb, which could have affected the test outcomes, were also excluded.

### 2.3. Instruments and Measures

Sociodemographic and health data were collected using a proprietary questionnaire created by the rehabilitation center, specifically for preliminary interviews and assessments during visits. The Oswestry Disability Index (ODI) was used to evaluate the degree of limitation in daily activities related to the disability of individuals experiencing lower back pain. The questionnaire consists of 10 questions, each with 6 possible responses rated on a scale from 0 to 5, depending on the extent to which the activity is limited by pain. To determine the intensity of lower back and phantom pain, a visual analog scale (VAS) was used. This scale allows for a subjective assessment of pain, consisting of a 10-point scale, where 0 represents no pain and 10 represents the worst pain the subject has ever experienced. The Amputee Mobility Predictor (AMPnoPRO) was used to assess motor function. This test, which requires minimal equipment, is a practical and reliable instrument [22]. It consists of 19 tasks performed without a prosthesis. The AMPnoPRO test was translated into Polish and back translated to ensure translation accuracy.

### 2.4. Study Procedures

Data were collected from existing medical records held at the Znowu w Biegu Rehabilitation Center. These included medical charts, surveys, questionnaires, patient tests, and interviews. Data consistency was ensured because all tests were conducted by qualified physiotherapists working in a single facility. The study received approval from the Ethics Committee of the Collegium Medicum, Jagiellonian University in Krakow (approval number: 118.0043.1.220.2024).

### 2.5. Statistical Analysis

Statistical analysis was performed using Statistica software (Version 13, StatSoft, Inc., Tulsa, OK, USA). The Shapiro–Wilk test was employed to assess the normality of distribution for all quantitative variables prior to further analysis. Variables with a normal distribution (*p* > 0.05) were presented as means and standard deviations, while those deviating from normality (*p* < 0.05) were reported as medians and interquartile ranges (IQRs). Group comparisons for normally distributed variables were performed using the Student’s *t*-test, whereas the Mann–Whitney U test was applied for non-normally distributed variables. Additionally, an analysis of covariance (ANCOVA) was performed. Qualitative variables were expressed numerically and presented as percentages for each category. These variables were compared using Fisher’s exact test because of the small size of the groups. Correlations between variables were analyzed using Spearman’s correlation coefficient. A significance level of *p* < 0.05 was adopted for all statistical analyses.

## 3. Results

### 3.1. Description of Study Groups

Study Group: The study group consisted of 15 men who were amputee football players. The average age of the group was 32.2 ± 8.6 years (*p* = 0.730), ranging from 18.0 to 49.0 years. The average weight was 77.3 ± 7.7 kg (*p* = 0.762), ranging from 60.0 to 89.0 kg. The average height was 1.8 ± 0.1 m (*p* = 0.915), ranging from 1.7 to 2.0 m. The mean BMI was 23.7 ± 2.7 (*p* = 0.484), with values ranging from 19.6 to 29.8. The median time since amputation was 144 (IQR: 264) months (*p* = 0.020). The median number of training sessions per week was 2 (IQR: 1) (*p* = 0.024), with the median duration of each session being 90 (IQR: 30) minutes (*p* < 0.001). Of the athletes, 73.3% had attended higher education, while the remainder had completed secondary education. In addition, 73.3% of players were professionally active. Among the participants, 33.3% reported comorbidities. Athletes with below-knee amputations made up 46.7% of the group, those with above-knee amputations 26.7%, and individuals with congenital limb defects 26.7%.

Control Group: The control group consisted of 15 men who did not participate in adapted sports. The average age of this group was 51.8 ± 14.6 years (*p* = 0.279), ranging from 20.0 to 70.0 years. The average weight was 85.0 ± 11.2 kg (*p* = 0.081), ranging from 68.0 to 100.0 kg. The average height was 1.8 ± 0.1 m (*p* = 0.832), ranging from 1.6 to 1.9 m. The mean BMI was 27.5 ± 4.5 (*p* = 0.501), with values ranging from 20.5 to 37.6. The median time since amputation was 18 (IQR: 14) months (*p* < 0.001). This group exhibited a lower level of physical activity, with only 80.0% engaging in any form of exercise, primarily at home. Among these men, 60.0% had completed secondary education, while the rest had attended higher or vocational education. In this group, 20.0% were professionally active. Among all participants, 60.0% had comorbidities. Men with above-knee amputations constituted 80.0% of the study group, whereas those with below-knee amputations accounted for 20.0%.

### 3.2. Characteristics of the Groups in Terms of Amputation

The majority of participants in the study group had below-knee amputations (46.7%), whereas in the control group, most individuals (80.0%) had above-knee amputations. The study group included individuals with congenital limb defects (26.7%), which were not observed in the control group, Amputation more commonly involved the right limb in both groups (53.3% and 60.0% in the study and control groups, respectively) (Table 1)

**Figure 1:**
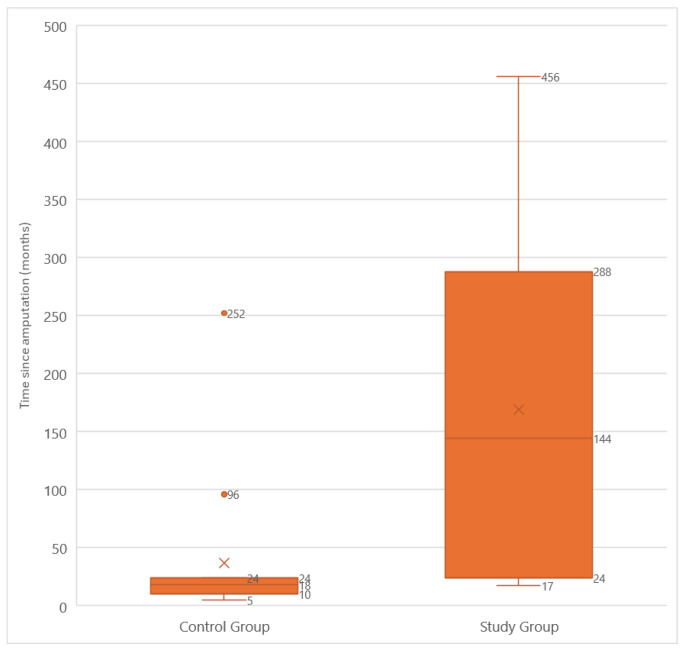
Time from limb amputation to the time of the study among the participants.

The reasons for amputation varied between the groups. The most common cause in the study group was trauma (33.3%), whereas in the control group, vascular disorders (33.3%) and trauma (33.3%) were equally prevalent (Table 1). The median time since amputation was 144 (IQR: 264) months in the study group and 18 (IQR: 14) months in the control group. A significantly shorter time since amputation was observed in the control group (*p* = 0.001) (Figure 1).

**Figure 2:**
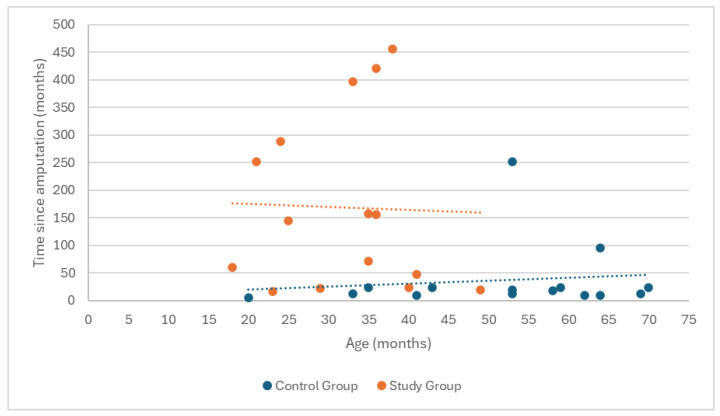
Correlation analysis between participants’ age and time since limb amputation.

Spearman’s correlation analysis of the relationship between age and time since amputation did not show a statistically significant relationship in either the study group (ρ = −0.05, *p* = 0.859) or the control group (ρ = 0.204, *p* = 0.465). These results suggest that age did not have a significant effect on the time elapsed since amputation, either among the amputee football players or in the control group (Figure 2).

### 3.3. Phantom Pain Characteristics

The analysis compared the occurrence of phantom pain between amputee football players and the control group. Phantom pain was reported by 53.3% of the study group and 66.7% of the control group. It is noteworthy that 60.0% of those in the control group who were not currently experiencing phantom pain had previously experienced phantom pain. Playing amputee football did not have a significant effect on the occurrence of phantom pain in the study group (*p* = 0.710). Furthermore, Spearman’s correlation did not reveal a statistically significant correlation between age and the occurrence of phantom pain in either group (study group: ρ = 0.077, *p* = 0.784; control group: ρ = 0.148, *p* = 0.599). Before amputation, 46.7% of players in the study group and 53.3% of those in the control group reported limb pain. Among those who experienced phantom pain after amputation, 72.2% experienced pain in the amputated limb prior to the procedure. A statistically significant relationship was found between preamputation pain and the occurrence of phantom pain after the procedure (*p* = 0.008), indicating that preoperative pain is a significant risk factor for phantom pain.

**Figure 3:**
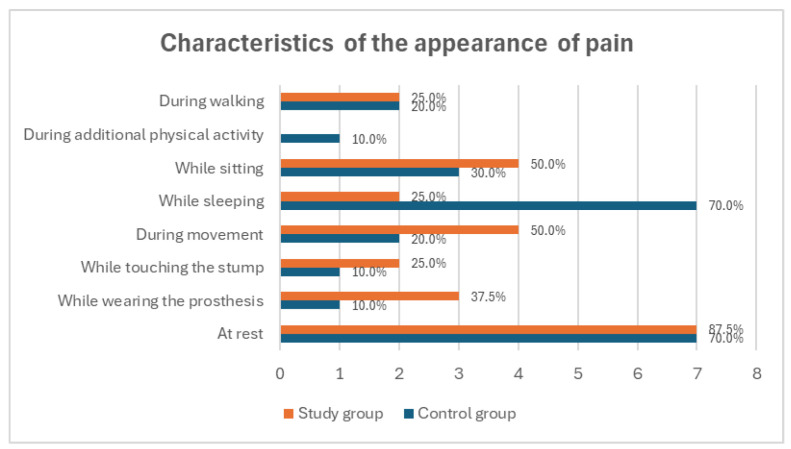
Summary comparison of activities during which study participants most frequently experienced phantom pain.

**Figure 4:**
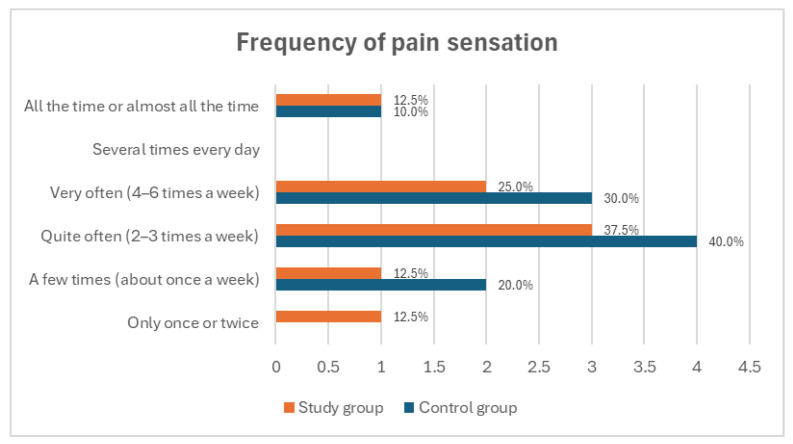
Comparison of the frequency of phantom pain occurrences among all study participants.

Analysis of pain intensity and characteristics revealed differences between the groups. In the study group, 87.5% of amputee football players experienced phantom pain at rest; however, no pain was reported during the additional physical activity. In the control group, pain most frequently occurred at rest and during sleep (70.0% each) and least frequently during prosthesis use, stump touching, and additional physical activity (10.0% each) (Figure 3). The largest proportion of participants in both groups experienced phantom pain 2–3 times per week (37.5% in the study group and 40.0% in the control group) (Figure 4). There was no statistically significant difference in the frequency of pain between amputee football players and individuals with lower limb amputations (*p* = 0.914), suggesting that playing football does not significantly influence the frequency of phantom pain symptoms.

**Figure 5:**
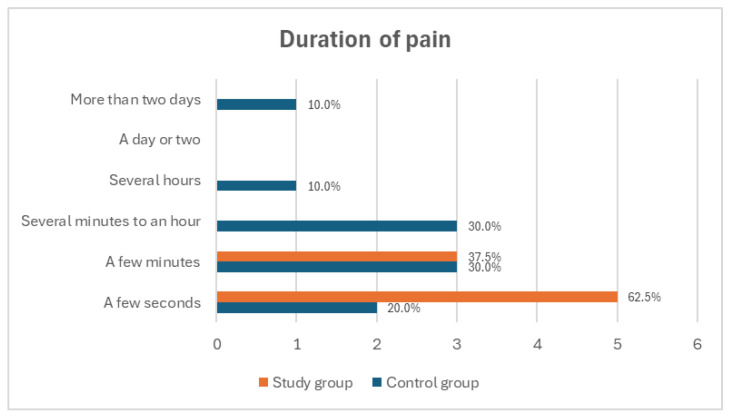
Average reported duration of phantom pain among study participants.

**Figure 6:**
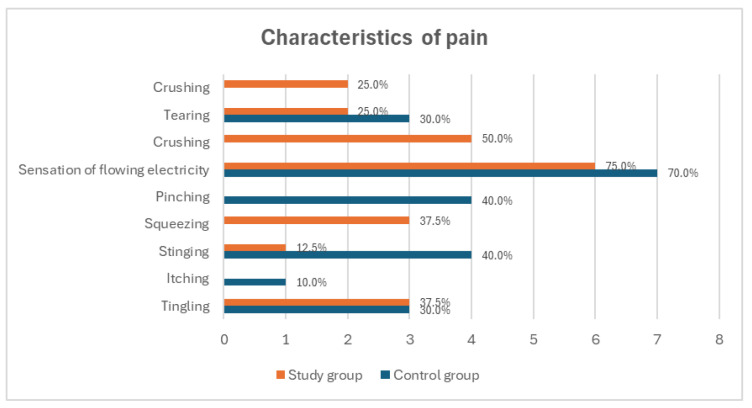
Summary comparison of the most frequently reported descriptions of phantom pain among study participants.

**Figure 7:**
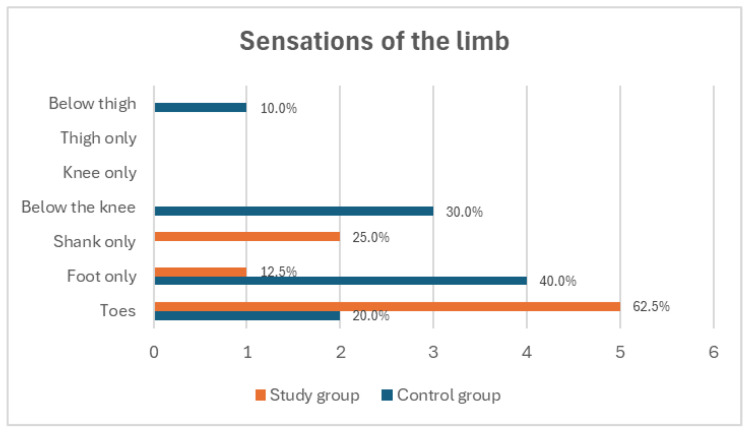
Presentation of the most commonly reported locations of phantom pain among study participants.

The duration of pain also differed between the groups; in the study group, pain most frequently lasted for a few seconds (62.5%), whereas in the control group, it most often persisted for several minutes (30.0%) or from several minutes to a few hours (30.0%) (Figure 5). Phantom pain in both groups was most commonly described as a sensation of flowing electricity (75.0% in the study group and 70.0% in the control group) (Figure 6). In the study group, pain occurred most often in the toes of the amputated limb (62.5%), whereas in the control group, it was predominantly reported in the foot (40.0%). No pain was reported in the knee or thigh regions in either group (Figure 7).

**Figure 8:**
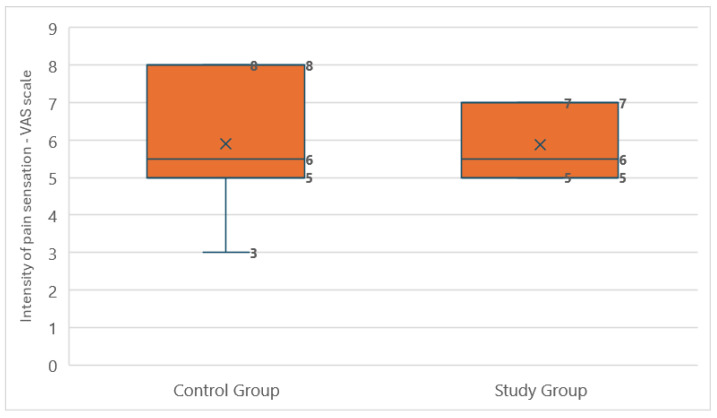
Comparison of the average phantom pain intensity reported on the visual analog scale between amputee football players and the control group.

The median intensity of phantom pain was 5.5 (IQR: 2) in the study group and 5.5 (IQR: 3) in the control group. Due to the lack of normal distribution in the study group (*p* = 0.006) and the presence of a normal distribution in the control group (*p* = 0.107), the Mann–Whitney U test was used for comparison. No statistically significant difference in pain intensity was found between the groups (*p* = 0.965), indicating that there was no basis for concluding that the players experienced pain of different intensities (Figure 8). Spearman’s correlation analysis of the relationship between time since amputation and the occurrence of phantom pain in the study group showed a negative correlation close to statistical significance (ρ = −0.495, *p* = 0.061), suggesting that phantom pain may decrease over time since amputation. No significant relationship was found in the control group (ρ = −0.316, *p* = 0.251), indicating no clear association between the time since amputation and phantom pain in this group.

Analysis of covariance (ANCOVA) revealed no significant differences in the level of phantom pain (on the VAS scale) between amputee football players and individuals in the control group after accounting for age, time since amputation, and the results of the AMPnoPRO functional test.

### 3.4. Lower Back Pain Characteristics

This study compared the frequency of lower back pain between amputee football players and a control group, revealing significant differences between these groups. In the control group, 86.7% of the participants reported lower back pain, whereas only 33.3% of amputee football players experienced such discomfort. Prior to amputation, 46.7% of the individuals in the control group reported lower back pain compared with only 6.7% in the study group. Statistical analysis showed a significant relationship between the groups, indicating that lower back pain occurred more frequently in those who did not participate in football (*p* = 0.008). However, Spearman’s correlation did not show a significant correlation between age and lower back pain in either group (study group: ρ = 0.131, *p* = 0.641; control group: ρ = 0.091, *p* = 0.746).

Among the amputee football players, lower back pain most commonly occurred during sitting and movement (60.0% each), whereas no pain was reported during additional physical activity or while playing amputee football. In the control group, back pain predominantly occurred while sitting (61.5%). The majority of individuals in the study group (60.0%) experienced pain 2–3 times per week, whereas in the control group, 46.2% of participants experienced pain once a week (Table 2). No statistically significant differences were found in the frequency of pain between the groups, suggesting that playing football did not significantly affect its intensity (*p* = 1.000).

**Figure 9:**
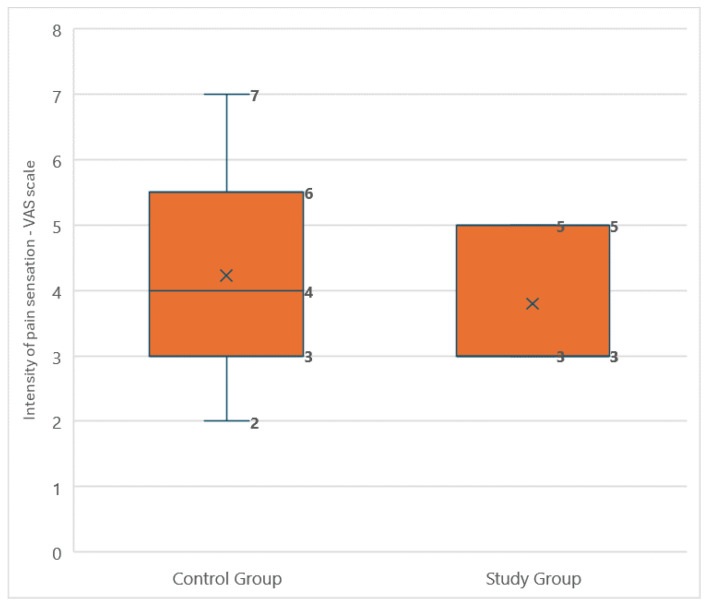
Comparison of the average lower back pain intensity reported on the visual analog scale between amputee football players and the control group.

The intensity of pain did not differ significantly between the groups. The median pain intensity was 3 (IQR: 2) for the amputee football players and 4 (IQR: 2) for the control group. The Shapiro–Wilk test indicated a non-normal distribution of pain intensity in the study group (*p* = 0.006), while the control group showed a normal distribution (*p* = 0.161). Consequently, the Mann–Whitney U test was applied for group comparison. The lack of significant differences (*p* = 0.767) suggests that both the player and control groups experienced pain of similar intensity (Figure 9). The results of the ANCOVA analysis did not reveal significant differences in lower back pain levels between the study groups, as measured by the VAS. Accounting for confounding factors such as age, time since amputation, BMI, ODI scores, and AMPnoPRO test results did not affect the statistical significance of the differences between the groups. Ultimately, lower back pain levels were comparable in both groups regardless of the variables analyzed.

**Figure 10:**
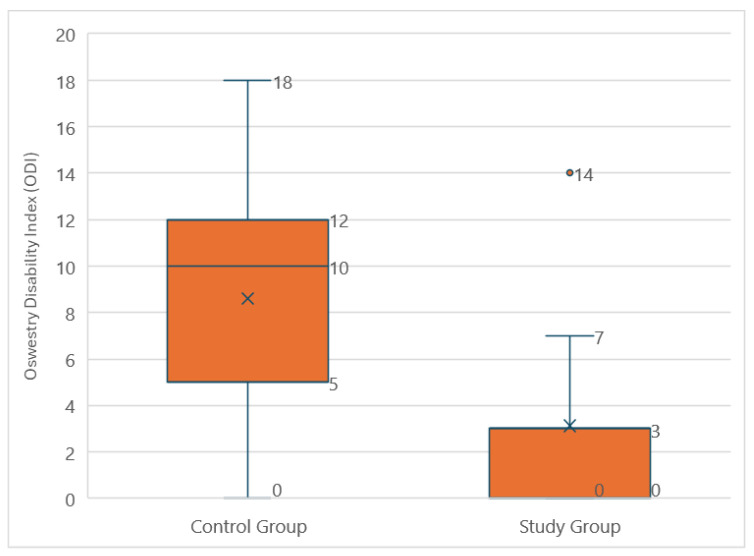
Comparison of the Oswestry Disability Index (ODI) scores from amputee football players and the control group.

Assessment of disability based on the Oswestry Disability Index (ODI) showed that 93.3% of individuals in the study group had minimal disability, while in the control group, 53.3% of participants had minimal disability. The remaining participants in the control group (46.7%) had moderate disability. The median ODI score was 3 (IQR: 3) in the study group and 10 (IQR: 7) in the control group (Figure 10). The Shapiro–Wilk test indicated a non-normal distribution of ODI scores in the study group (*p* < 0.001), while the control group showed a normal distribution (*p* = 0.361). Given this, the Mann–Whitney U test was used to compare the groups. This difference was statistically significant (*p* = 0.006), indicating greater limitations in daily activities among those who did not participate in amputee football.

ANCOVA revealed significant differences in the level of disability between the amputee football players and the control group, accounting for variables such as age, time since amputation, BMI, and AMPnoPRO test scores. After adjusting for age, the original difference in disability levels favoring amputee football players was no longer statistically significant. This result suggests that the lower level of disability in players may be partly due to their younger age and physical conditioning. When the time since amputation was considered, the difference between the groups decreased from 5.5 to 4.1 points but remained statistically significant, indicating that amputee football players still had an advantage in functional abilities, despite the time elapsed since amputation. In the analysis adjusted for BMI, the difference in disability levels decreased from 5.5 to 5.4 points in favor of the study group. This result suggests that amputee football players exhibit better functional indicators regardless of body weight. However, after accounting for AMPnoPRO test scores, the original difference between the groups was not statistically significant. This may indicate that a higher level of motor fitness among amputee football players contributes to lower levels of disability. Additionally, correlation analysis of the relationship between the Oswestry Disability Index (ODI) and time since amputation did not reveal a statistically significant relationship in either group. In the amputee football group, Spearman’s correlation indicated a correlation of −0.418 (*p* = 0.122), suggesting a tendency toward a lower level of disability with more time since amputation, although this effect was not statistically significant. In the control group, the correlation was −0.147 (*p* = 0.601), indicating no significant relationship between time since amputation and the ODI score.

### 3.5. Physical Activity Characteristics

**Figure 11:**
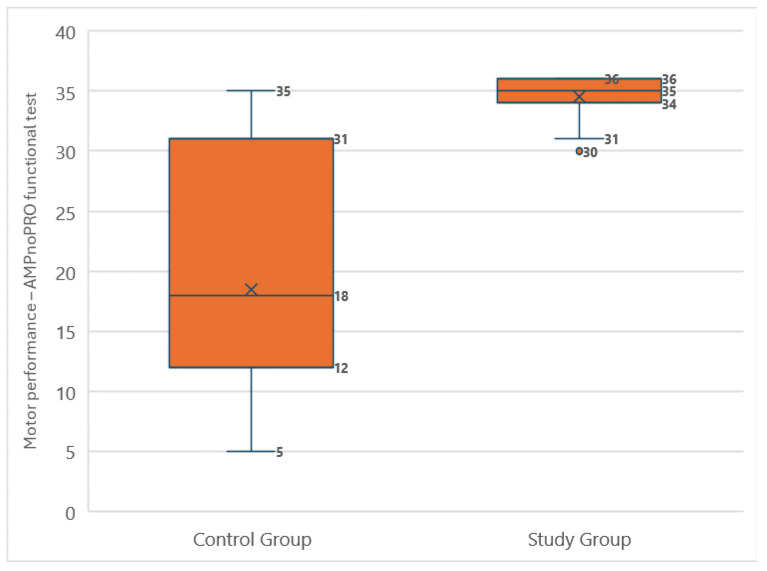
Comparison of AMPnoPRO functional test results between participants who played amputee football and the control group.

The analysis of motor fitness, assessed using a functional test, revealed significant differences between amputee football players and individuals with lower limb amputations who did not play football. The median score in the amputee football group was 35 (IQR: 2), whereas that in the control group was only 18 (IQR: 19). The Shapiro–Wilk test showed that the study group’s scores did not follow a normal distribution (*p* = 0.001), while the control group’s scores were normally distributed (*p* = 0.124). Consequently, the Mann–Whitney U test was used for comparison. The difference was statistically significant (*p* < 0.001), clearly indicating better motor fitness among the amputee football players (Figure 11). ANCOVA analysis, which accounted for age as a confounding factor, confirmed the significant difference between the groups, although the initial difference of 16.1 points decreased to 9.7 points after adjusting for age. This confirms that amputee football players achieve better motor performance, even when age differences are considered. The most common difficulty, both among amputee football players (46.7%) and in all participants from the control group (100.0%), was maintaining balance on the non-amputated leg for 30 s with eyes closed.

**Figure 12:**
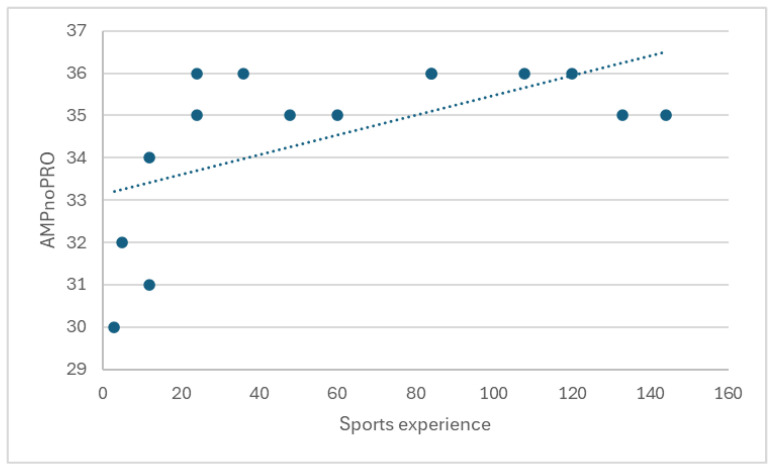
Presentation of the correlation between the average training duration in amputee football and motor performance, measured by the AMPnoPRO test.

Spearman’s correlation analysis revealed a statistically significant relationship between training duration in amputee football and motor performance, measured by the AMPnoPRO test, with a coefficient of ρ = 0.602 (*p* = 0.017). This finding suggests a moderately positive correlation, indicating that a longer training period is associated with better motor test results (Figure 12).

**Figure 13:**
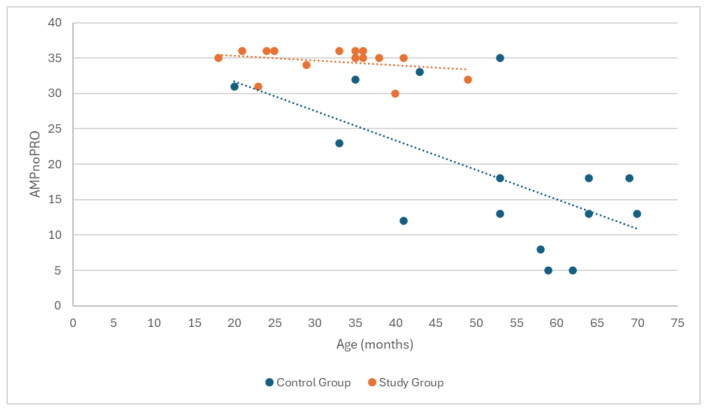
Presentation of the correlation between participants’ age and motor performance, measured by the AMPnoPRO test.

Spearman’s correlation analysis did not show a statistically significant relationship between age and functional test results in amputee football players (ρ = −0.330, *p* = 0.230). However, in the control group, a near-significant relationship was observed between age and functional test results (ρ = −0.467, *p* = 0.080), suggesting a potential trend in which older age may negatively impact performance, although this result did not reach the threshold for statistical significance (Figure 13). In both groups, no significant relationships were found between motor performance and place of residence or education, indicating that these factors did not have a meaningful impact on the functional test results.

An analysis of covariance (ANCOVA) comparing motor performance levels between amputee football players and the control group while accounting for the time since amputation, BMI, and ODI test results, revealed a significant difference between the groups. The initial difference in motor performance (16.1 points in favor of the study group) decreased to 14.1 points after adjusting for time since amputation, increased to 17.1 points after adjusting for BMI, and decreased to 14.4 points after adjusting for ODI test results. Nonetheless, amputee football players consistently achieved better motor performance than did the control group. The results confirm that amputee football players have a motor performance advantage regardless of the variables analyzed.

## 4. Discussion

In this retrospective study, we conducted a comparative analysis of lower back pain and phantom limb pain in individuals post-amputation, comparing them with amputee football players, and compared the motor performance of both groups. The results of our study revealed that amputee football players demonstrated significantly better motor performance, and that a longer training period correlated with higher motor test scores. Furthermore, our analysis showed that preoperative limb pain was a significant risk factor for phantom pain. We also observed that lower back pain was more prevalent among individuals who did not participate in football and was associated with greater difficulties in daily functioning.

Based on the AMPnoPRO test, the results of our study indicate that amputee football players exhibit significantly better motor skills, which may translate to increased proficiency in performing daily activities. In the control group, a trend was observed suggesting that older age may negatively affect functional test outcomes, although this relationship was not statistically significant. These observations are consistent with those of previous studies that demonstrated the beneficial effects of football and adaptive sports on motor skill development. Research conducted by Yazicioglu et al. [23] showed that participation in football training significantly improved locomotor abilities and balance in amputee football players, highlighting the positive impact of adaptive sports on motor skill development in this group. Monteiro et al. [24] confirmed these findings, noting that amputee football players achieve higher scores in terms of functional and social abilities, suggesting that regular physical activity has a positive effect on the quality of life for individuals with amputations. Similarly, Guchan et al. [25] conducted studies comparing endurance and physical fitness and demonstrated the superiority of amputee football players in anaerobic capacity, balance, and overall motor skills. Although Yazicioglu and Guchan’s studies did not record significant differences in motor skills assessed using the Berg Balance Scale, the results suggest that amputee football may be a beneficial form of rehabilitation for individuals with amputation. Mikami et al. [26] also confirmed these findings by comparing the endurance of amputee football players with that of healthy men, showing that their fitness levels are comparable and that participation in adaptive sports can help maintain high physical fitness despite amputations.

Pain in the lumbar spine is a common ailment in the general population and complicates the examination of the relationship between these issues and prior amputation. Our results confirmed that lower back pain may be associated with amputation and is significantly more frequent in sedentary individuals, including those who do not play football. In contrast, no lower back pain was observed in amputee football players either during additional physical activities or while playing football. This may be due to the fact that this sport engages various motor patterns that not only develop balance and endurance but also strengthen the muscles stabilizing the spine. These findings are consistent with those of Sadowski et al. [12] and Sivapuratharasu et al. [27], who confirmed a clear link between lower back pain and a history of amputation. Sadowski et al. [12] conducted comparative analyses of the frequency of these issues in the general population and showed that back pain is more common in individuals with amputations than in healthy individuals with similar pain perception parameters. Their study also revealed that middle-aged individuals who have a sedentary lifestyle and do not engage in additional physical activity experience pain more frequently. Similar conclusions were drawn by Wasser et al. [28], who observed that physical activity had a positive effect on reducing lower back pain.

The results of our study also indicated significantly greater limitations in daily activities among individuals in the control group than among amputee football players, as assessed using the Oswestry questionnaire. An ANCOVA confirmed these differences, showing that the lower level of disability among amputee football players may be attributed to their younger age, better motor skills, and training. Despite the passage of time since amputation and differences in BMI, the players still demonstrated superior functional abilities. Lower disability scores in the player group suggest that participation in sports may contribute to improved functional independence and QoL. These observations are consistent with the findings of Butowicz et al. [29], who revealed a high level of disability among individuals with unilateral lower limb amputation who experienced lower back pain. This supports the hypothesis that engagement in physical activity offers positive psychosocial and physical benefits to individuals with amputation.

The analysis did not show any impact of football on the frequency or intensity of phantom pain compared to individuals who did not engage in additional physical activity. However, phantom pain occurred more frequently during rest, suggesting that its severity may be associated with lack of movement. Ross et al. [30] indicated that phantom pain occurred in 84.3% of athletes participating in sports such as sled hockey, golf, cycling, and running, with moderate intensity. Although the frequency of pain was high, the analysis did not reveal a significant association between the frequency of sports participation and pain intensity, suggesting that adaptive sports may lead to more frequent unpleasant sensations but does not necessarily affect their severity. It has also been established that participation in adaptive sports does not increase phantom pain intensity. Additionally, Bragaru et al. [31] emphasized that sports activities can effectively contribute to the alleviation of phantom pain. However, Grobler et al. [32] pointed out that Paralympic athletes often use more painkillers and experience a greater number of injuries and chronic pain than able-bodied athletes, highlighting the need for further research on the impact of adaptive physical activity on reducing phantom pain.

This study indicates a relationship between preoperative pain and the occurrence of phantom pain following amputation. Individuals who experienced pain in the lower limb prior to surgery had an increased risk of developing phantom pain compared with those who did not experience pain before amputation. Yin et al. [33] demonstrated that limb pain associated with an ongoing disease prior to amputation significantly increases the risk of phantom pain. Similar results were obtained by Griffin et al. [34], who observed a higher frequency and intensity of phantom pain in patients with preoperative pain.

These findings are important for developing effective rehabilitation programs, especially in the context of pain management, and for improving motor function and quality of life in individuals after amputation. Studies suggest that physical activity, including participation in adaptive sports such as amputee football, can play a crucial role in reducing pain and supporting rehabilitation. To reduce the risk of future pain, it is essential to implement pain management strategies prior to amputation. Tailoring training programs to the individual needs of patients is particularly important, as lack of physical activity and social isolation can negatively impact health. Adaptive sports can not only support the development of motor and social skills but also contribute to reducing the severity of phantom pain and lower back pain, which may result in a decreased level of disability. Therefore, early implementation of rehabilitation and physical activity is key to managing pain and improving quality of life in the initial stages of amputation.

One of the key strengths of this study is its focus on a unique group, including athletes with amputations and individuals with amputations, for whom there are limited available data. This study makes a significant contribution to the literature, particularly in the area of adaptive sports such as amputee football, which has not been sufficiently studied. Additionally, the use of well-established measurement tools, such as the AMPnoPRO test and the Oswestry Disability Index, enhances the reliability of our findings.

However, our study has several important limitations. First, the small size of the study groups, due to the limited availability and uniqueness of the population, restricts the generalizability of the results. Further research with a larger sample size is necessary to confirm these conclusions. Moreover, differences in age and BMI among the participants may have influenced the results, making it difficult to attribute the observed effects solely to participation in football.

Another limitation was the reliance on self-reported pain by the participants, which could have led to data distortion. Future studies should incorporate more-objective measures of pain and physical activity to improve the precision of results. The lack of randomization in our study presents an additional challenge as it may lead to the influence of uncontrolled confounding variables. Randomized controlled trials should be conducted to better control for these factors and increase the credibility of the findings.

Additionally, the retrospective data collection method limits the analysis, particularly in the context of psychological and social factors. Future research should include an assessment of mental health, social support, and motivation to gain a more comprehensive understanding of how these factors influence pain, motor performance, and disability levels in amputated individuals.

Future studies should focus on the long-term effects of sports participation on the physical and psychological outcomes of individuals post-amputation. It is also important to investigate the impact of different adaptive sports on individuals’ health to identify the specific benefits of each discipline. Such research could provide valuable insights into optimizing rehabilitation programs, allowing for better alignment of sports activities with the individual needs of patients.

## 5. Conclusions

This study showed that amputee football participants have better motor performance and lower back pain than non-participants in adaptive sports. While amputee football seems to be linked to improved functional outcomes, these differences may partly result from athletes’ younger age and higher baseline fitness. Further research with larger sample sizes and randomized controlled designs is required to establish a causal relationship. Additionally, adaptive sports, such as amputee football, can be valuable in rehabilitation, offering physical and social benefits that enhance quality of life for individuals post-amputation.

## Figures and Tables

**Table 1:** Baseline characteristics.

	Study Group (n = 15)	Control Group (n = 15)
Age (years)		
Mean ± SD	32.2 ± 8.6	51.8 ± 14.6
*p*-value	<0.001	
Weight (kg)		
Mean ± SD	77.3 ± 7.7	85.0 ± 11.2
*p*-value	0.038	
Height (m)		
Mean ± SD	1.8 ± 0.1	1.8 ± 0.1
*p*-value	0.123	
BMI (kg/m^2^)		
Mean ± SD	23.7 ± 2.7	27.5 ± 4.5
*p*-value	0.001	
Type of amputation		
Foot amputation	0 (0.0%)	0 (0.0%)
Below-knee amputation	7 (46.7%)	3 (20.0%)
Knee disarticulation	0 (0.0%)	0 (0.0%)
Above-knee amputation	4 (26.7%)	12 (80.0%)
Hip disarticulation	0 (0.0%)	0 (0.0%)
Hemipelvectomy	0 (0.0%)	0 (0.0%)
Congenital defect	4 (26.7%)	0 (0.0%)
Side of the amputated limb		
Right	8 (53.3%)	9 (60.0%)
Left	7 (46.7%)	6 (40.0%)
Reason for amputation		
Vascular	4 (26.7%)	5 (33.3%)
Diabetic	0 (0.0%)	1 (6.7%)
Traumatic	5 (33.3%)	5 (33.3%)
Cancer	2 (13.3%)	2 (13.3%)
Congenital defect	4 (26.7%)	0 (0.0%)
Septic	0 (0.0%)	2 (13.3%)

SD—standard deviation, BMI—body mass index.

**Table 2:** Characteristics of lower back pain.

Tested Feature	Study Group (n = 15)	Control Group (n = 15)
Characteristics of the appearance of pain		
At rest	1 (20.0%)	2 (15.4%)
During movement	3 (60.0%)	6 (46.2%)
During additional physical activity	0 (0.0%)	6 (46.2%)
During work	0 (0.0%)	1 (7.7%)
While playing amputee football	0 (0.0%)	0 (0.0%)
While wearing the prosthesis	1 (20.0%)	4 (30.8%)
While sitting	3 (60.0%)	8 (61.5%)
After waking up	2 (40.0%)	2 (15.4%)
Frequency of pain sensation		
Only once or twice	1 (20.0%)	0 (0.0%)
A few times (about once a week)	0 (0.0%)	6 (46.2%)
Quite often (2–3 times a week)	3 (60.0%)	4 (30.8%)
Very often (4–6 times a week)	0 (0.0%)	2 (15.4%)
Several times every day	1 (20.0%)	1 (7.7%)
All the time or almost all the time	0 (0.0%)	0 (0.0%)

## Data Availability

The data that support the findings of this study are available upon reasonable request.
